# AUXILIARY THREAD TO TIE A SHORT THREAD OF A RUNNING SUTURE

**DOI:** 10.1590/0102-672020180001e1469

**Published:** 2019-12-20

**Authors:** Andy PETROIANU

**Affiliations:** 1 Medical School, Federal University of Minas Gerais, Belo Horizonte, MG, Brazil

**Keywords:** Sutures, Suture techniques, Suture anchors, Suturas, Técnicas de sutura, Âncoras de sutura

## INTRODUCTION

When finishing a continuous suture, often a thread segment is too short to be tied. In this situation, the knots are made with the aid of needle holders or hemostatic forceps, but the result may be unsatisfactory^1^ ^,^ ^2^ ^,^ ^3^. Another option is to fix it with another thread, which is tied with the short thread, increasing the cost of the operation.

The use of an auxiliary thread to facilitate the making the end knot of a continuous short-threaded suture has been used by the author for over 40 years with good results. It is likely that other surgeons have also discovered this tactic and use it, but the author has not found published information and has only disclosed it personally during the operative acts.

## TECHNIQUE

At the end of a continuous suture, if the distal remnant is too short to be tied, an unpunched thread is passed through the last suture loop. It can be any available thread segment that is in operative field to be tied to support the knot tension and should have at least 10 cm long.

**FIGURE 1:**
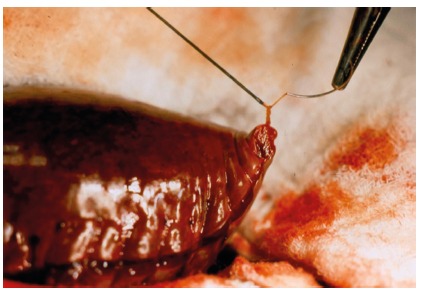
Auxiliary thread for tying short suture in continuous suture: 2-0 silk thread, pierced at the end of a continuous suture with 3-0 chrome catgut thread, for splenic capsule synthesis after subtotal splenectomy. It can be observed the knots that were made with the short catgut thread. Then the silk auxiliary thread is easily removed.

After passing through the last loop of the continuous suture, the auxiliary suture is folded over and pulled enough so that the continuous suture loop can be tied with the small distal remnant of the suture (Figure 1). Once the knot is completed, the auxiliary thread is removed or, if the continuous suture thread is extremely short, it may be included as part of the knot.

## DISCUSSION

The role of the auxiliary thread resembles that of a catalyst, it enters the thread to be tied, facilitates giving the knot and, at the end, it get out in same that have got in.

This technique has been used by the author for over 40 years, without prior publication and always with good results.
